# Supplementary material for: Task-dependent fractal patterns of information processing in working memory
Source: Sci Rep. 2022 Oct 25;12:17866. doi: 10.1038/s41598-022-21375-1 (PMC9596406; doi:10.1038/s41598-022-21375-1)
Supplement: Supplementary file 1 — Supplementary Information 1. [file 41598_2022_21375_MOESM1_ESM.pdf]

Supplementary Materials to *Task-dependent fractal patterns of information processing in working memory*

Jeremi K. Ochab[1,2,*], Marcin Wątorek[3,1], Anna Ceglarek[4], Magdalena Fąfrowicz[4], Koryna Lewandowska[4], Tadeusz Marek[4], Barbara Sikora-Wachowicz[4], and Paweł Oświęcimka[5,1,]**

[1]Institute of Theoretical Physics, Jagiellonian University, 30-348 Kraków, Poland
[2]Mark Kac Complex Systems Research Centre, Jagiellonian University, 30-348 Kraków, Poland
[3]Faculty of Computer Science and Telecommunications, Cracow University of Technology, 31-155 Kraków, Poland
[4]Department of Cognitive Neuroscience and Neuroergonomics, Jagiellonian University, 30-348 Kraków, Poland
[5]Complex Systems Theory Department, Institute of Nuclear Physics, Polish Academy of Sciences, 31-342 Kraków, Poland
[*]jeremi.ochab@uj.edu.pl
[**]pawel.oswiecimka@ifj.edu.pl

ABSTRACT

We applied detrended fluctuation analysis, power spectral density, and eigenanalysis of detrended cross-correlations to investigate fMRI data representing a diurnal variation of working memory in four tasks: two visual-verbal and two nonverbal. We show that the degree of fractal scaling is regionally dependent on the engagement in cognitive tasks. A particularly apparent difference was found between memorisation in verbal and nonverbal tasks. Moreover, the detrended cross-correlations between brain areas were predominantly indicative of differences between the resting state and other tasks, between memorisation and retrieval, and between verbal and nonverbal tasks. The fractal and spectral analyses presented in our study are consistent with previous research related to visuospatial and verbal information processing, working memory (encoding and retrieval), and executive functions, but they were found to be more sensitive than Pearson correlations and showed potential for obtaining other subtler results. We conclude that regionally dependent cognitive task engagement can be distinguished based on the fractal characteristics of BOLD signals and their detrended cross-correlation structure.

A. Supplementary Figures

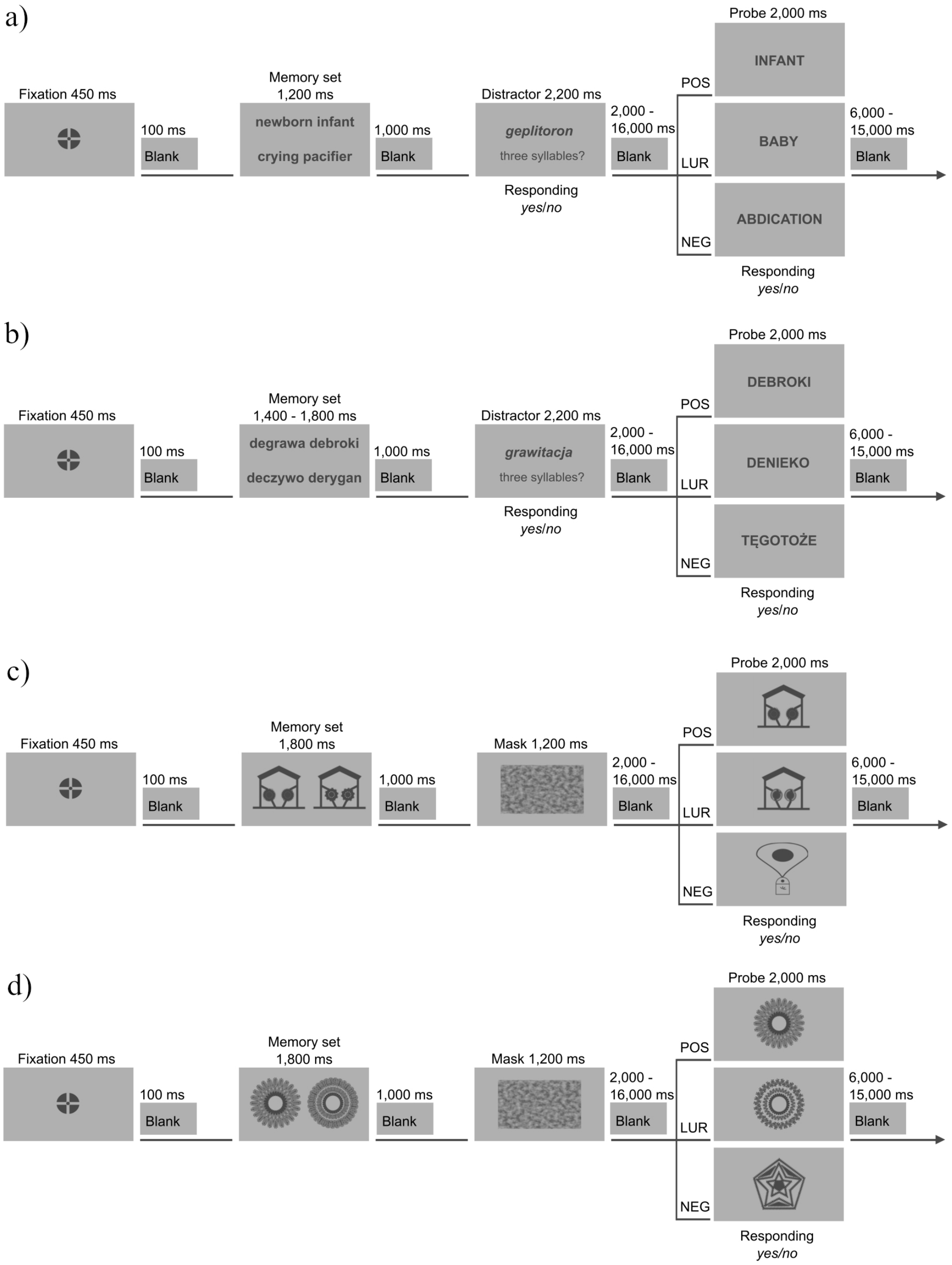

Figure A.1. Experimental tasks: a) semantic task, SEM; b) phonological task, PHO; c) local information processing task, LOC; d) global information processing task, GLO. All words and pseudo-words were presented in Calibri 22-point font. Stimuli were presented in dark gray (RGB 72, 72, 72) on a light gray (RGB 176, 176, 176) background, made with Inkscape.[1] The masks were generated with MATLAB.[2]

[1] Inkscape. Version 0.92.3. 2018. Inkscape Project. URL:https://inkscape.org/release/inkscape-0.92.3/
[2] MATLAB. Version 9.1.0 (R2016b). 2016. Natick, Massachusetts: The MathWorks Inc.

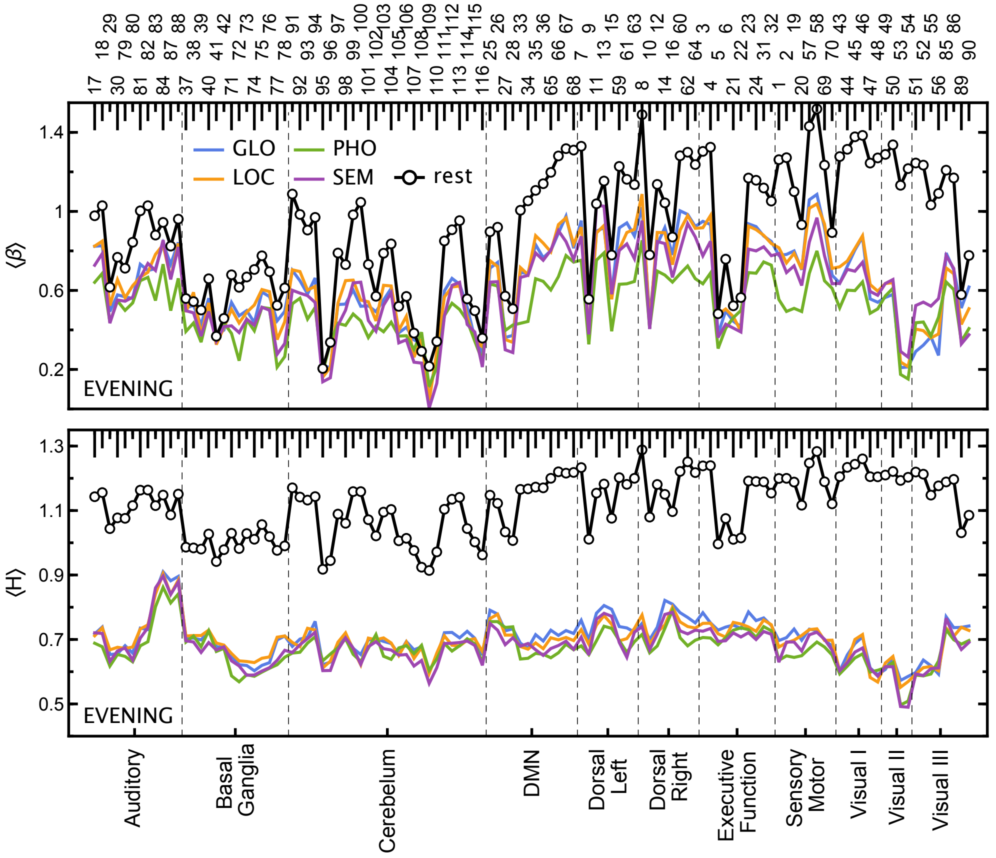

Figure A.2. Plots of the spectral, β, and Hurst, H, scaling exponents estimated for the entire time series in evening. The exponents were calculated for each ROI and ordered on the plot according to the AAL atlas (top) and resting-state networks (RSN). Abbreviations: visual-verbal tasks (semantic, SEM, and phonological, PHO), visual-nonverbal tasks (local information processing, LOC, and global information processing, GLO), resting-state (rest).

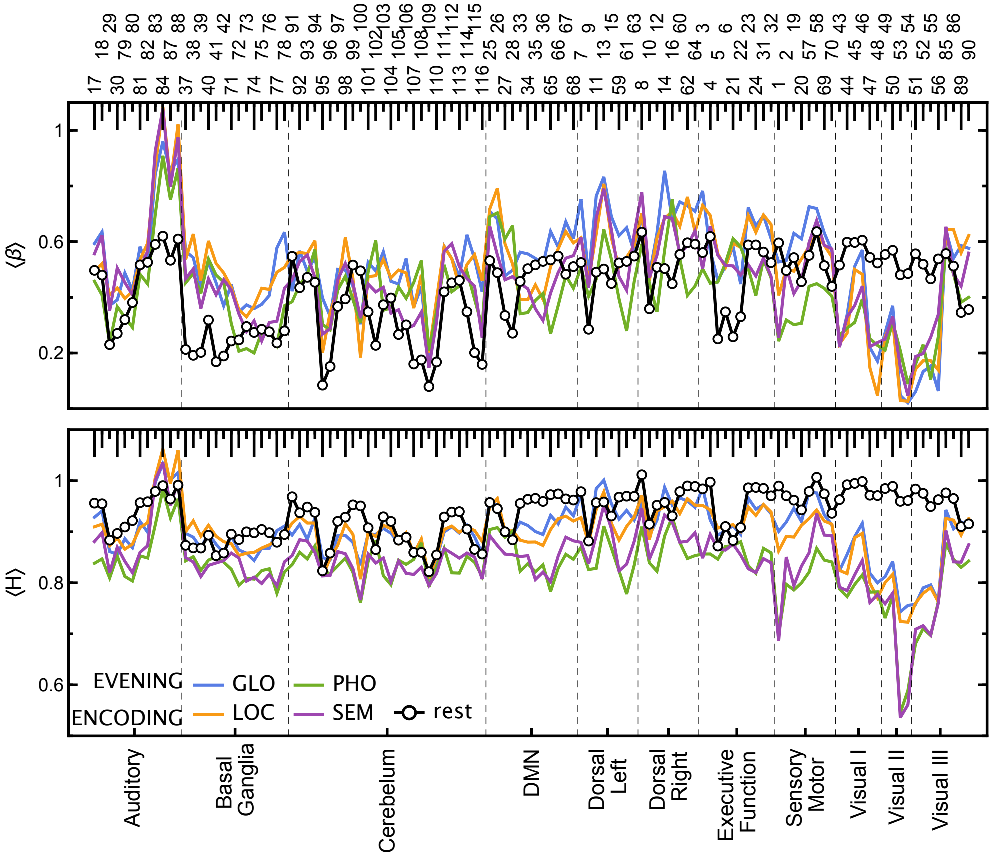

Figure A.3. Plots of the spectral, β, and Hurst, H, scaling exponents estimated for the encoding phase in evening. The exponents were calculated for each ROI and ordered on the plot according to the AAL atlas (top) and resting-state networks (RSN). Abbreviations: visual-verbal tasks (semantic, SEM, and phonological, PHO), visual-nonverbal tasks (local information processing, LOC, and global information processing, GLO), resting-state (rest).

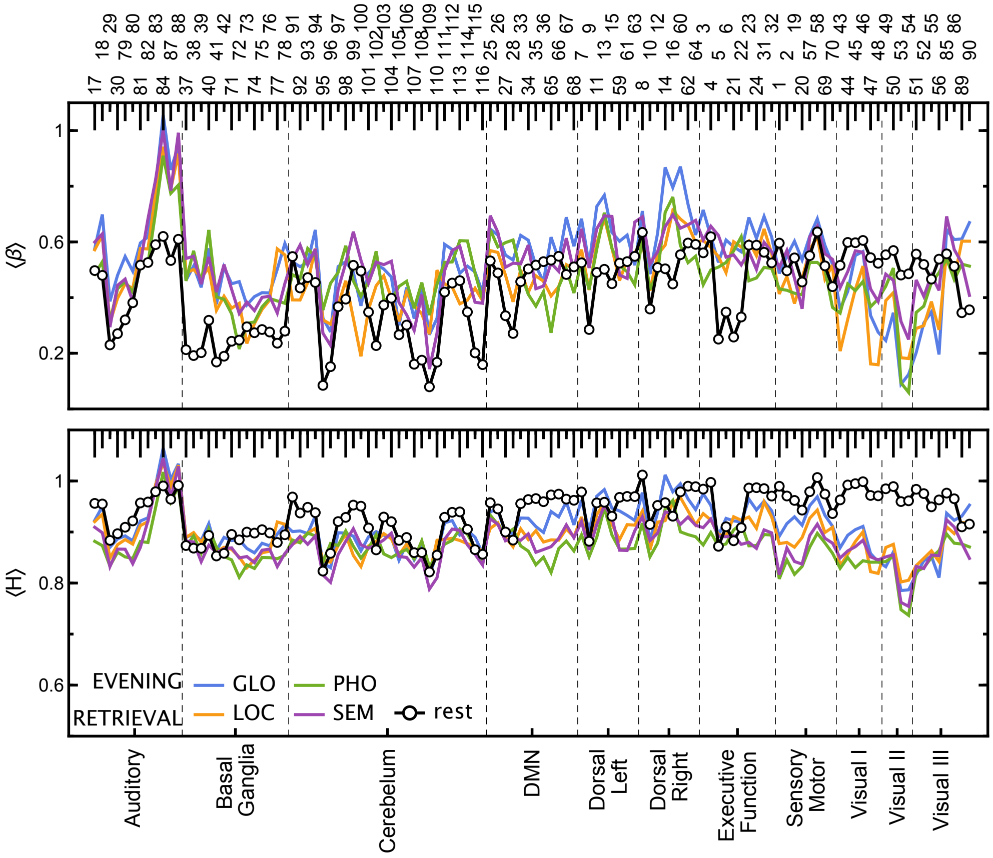

Figure A.4. Plots of the spectral, β, and Hurst, H, scaling exponents estimated for the retrieval of memories phase in evening. The exponents were calculated for each ROI and ordered on the plot according to the AAL atlas (top) and resting-state networks (RSN). Abbreviations: visual-verbal tasks (semantic, SEM, and phonological, PHO), visual-nonverbal tasks (local information processing, LOC, and global information processing, GLO), resting-state (rest).

B. Supplementary Tables

tasks/type of probe	positive	lure	negative
proportion of correct responses			
GLO	0.81 (0.16)	0.64 (0.18)	0.98 (0.10)
LOC	0.80 (0.15)	0.80 (0.18)	0.96 (0.14)
PHO	0.72 (0.16)	0.69 (0.19)	0.95 (0.13)
SEM	0.85 (0.15)	0.89 (0.14)	0.97 (0.12)
reaction times [ms]			
GLO	1245 (224)	1335 (209)	975 (163)
LOC	1319 (238)	1301 (188)	1065 (155)
PHO	1280 (226)	1414 (262)	1163 (230)
SEM	1057 (180)	1188 (221)	1068 (207)

Table B.1. Behavioural data for four types of tasks. The table presents the means (standard deviations) of the proportion of correct responses and reaction times for three types of probes: positive, lure, and negative.

		ENC	RET	REST
λ_1	RET			-1.8*
λ_2	ENC			1.8*
λ_3	RET			-1.7**
λ_4	ENC			3.8***
	RET			3.3***
λ_6	ENC			-2.0***
	RET			-1.7***
λ_8	RET			1.2*

Table B.2. Pairwise differences between eigenvalues of Pearson correlation matrices in phases ENC, RET and REST. Only statistically significant results are shown. Significance codes: p<0.001 '***', 0.01 '**', 0.05 '*'.

		GLO	LOC	PHO	SEM	REST
λ_5	PHO					-2.5***
	SEM					-1.7**
λ_{11}	GLO			0.94***		
	LOC			1.1***		
	PHO					-1.3***
	SEM					-0.91***
λ_{13}	GLO					-0.98*
	LOC					-1.1**
	PHO					-1.2***
	SEM					-1.5***

Table B.3. Pairwise differences between eigenvalues of Pearson correlation matrices in tasks GLO, LOC, PHO, SEM, and REST. Only statistically significant results are shown. Significance codes: p<0.001 '***', 0.01 '**', 0.05 '*'.

		ENC	RET	REST
λ_1	ENC		2.8***	7.6***
	RET			4.8***
λ_3	ENC		2.0**	3.0***
λ_{15}	ENC			-0.39***
	RET			-0.30**

Table B.4. Pairwise differences between eigenvalues of $\rho(q,s)$ correlation matrices in phases ENC, RET and REST. Only statistically significant results are shown. Significance codes: p<0.001 '***', 0.01 '**', 0.05 '*'.

		GLO	LOC	PHO	SEM	REST
λ_1	GLO				-4.3**	4.5***
	LOC				-4.8***	4.0**
	PHO					6.6***
	SEM					8.8***
λ_2	GLO				3.4*	
	LOC					-3.7**
	PHO					-4.0***
	SEM					-4.6***
λ_3	GLO					3.0**
	LOC					
	PHO					2.7*
	SEM					
λ_4	LOC			2.7*		4.5***
	SEM					2.6*
λ_5	LOC			-3.2***		
λ_7	GLO				-1.4*	
λ_9	GLO			-1.8***		
	PHO					1.7***
λ_{10}	GLO					-1.1*
λ_{15}	SEM					-0.43**

Table B.5. Pairwise differences between eigenvalues of $\rho(q,s)$ correlation matrices in tasks GLO, LOC, PHO, SEM, and REST. Only statistically significant results are shown. Significance codes: p<0.001 '***', 0.01 '**', 0.05 '*'.

eigenvalue	morning-evening
λ_3	-0.90*
λ_6	-0.74*
λ_8	-0.77*

Table B.6. List of eigenvalues of Pearson correlation matrices for which the effect of time of day was significant according to Scheffe's test. Significance codes: p<0.001 '***', 0.01 '**', 0.05 '*'.

eigenvalue	morning-evening
λ_3	1.16*
λ_9	0.58*

Table B.7. List of eigenvalues of $\rho(q,s)$ correlation matrices for which the effect of time of day was significant according to Scheffe's test. The baseline is resting state in the morning. Significance codes: p<0.001 '***', 0.01 '**', 0.05 '*'.

C. Supplementary Statistics: Code and Test Results

```
library(lme4)
summary(model.01 <- lmer(hurst ~ condition*task+task:mode+task:mode:condition
    + (1|ROI), data=df))
drop1(model.01, test="Chisq")
# Single term deletion result:
#                    npar     AIC     LRT Pr(Chi)
#<none>                  -8560.5
#condition:task:mode    4 -8564.7 3.8128  0.4319

summary(model.02 <- update(model.01, ~.- task:mode:condition))
drop1(model.02, test="Chisq")
# Single term deletion result:
```

```
#                npar     AIC   LRT   Pr(Chi)
#<none>              -8564.7
#condition:task    4 -8551.7  21.0 0.0003161 ***
#task:mode         4 -8209.8 362.9 < 2.2e-16 ***
```

Listing 1. R code for computing a mixed linear model for the global effects of time of day, task, task phase and their interactions, with ROIs as random effects and the Hurst exponent, H, as dependent variable. We performed a stepwise model selection by dropping single terms based on χ^2 tests.

Random effects:

Groups	Name	Variance	Std.Dev.
ROI	(Intercept)	0.0017170	0.04144
Residual		0.0007784	0.02790
Number of obs: 2086, groups:	ROI, 116		

Fixed effects:

Estimate Std. Error t value			
(Intercept)	0.9361674	0.0046380	201.846
conditionmorning	-0.0003843	0.0036633	-0.105
taskGLO	-0.0267674	0.0034267	-7.811
taskLOC	-0.0426949	0.0034267	-12.459
taskPHO	-0.0665273	0.0034267	-19.414
taskSEM	-0.0590249	0.0034278	-17.220
conditionmorning:taskGLO	-0.0011522	0.0044866	-0.257
conditionmorning:taskLOC	-0.0030608	0.0044866	-0.682
conditionmorning:taskPHO	0.0120013	0.0044866	2.675
conditionmorning:taskSEM	0.0043060	0.0044903	0.959
taskGLO:modeencoding	-0.0091881	0.0025904	-3.547
taskLOC:modeencoding	0.0010172	0.0025904	0.393
taskPHO:modeencoding	-0.0381264	0.0025904	-14.719
taskSEM:modeencoding	-0.0334943	0.0025960	-12.902

Table C.1. Output of the mixed linear model for the global effects of time of day, task, task phase and their interactions, with ROIs as random effects and the Hurst exponent, as computed in Listing 1.
